# The bronchiolitis epidemic in 2021–2022 during the SARS-CoV-2 pandemic: experience of a third level centre in Northern Italy

**DOI:** 10.1186/s13052-023-01425-8

**Published:** 2023-02-21

**Authors:** Martha Caterina Faraguna, Irene Lepri, Antonio Clavenna, Maurizio Bonati, Chiara Vimercati, Debora Sala, Alessandro Cattoni, Maria Luisa Melzi, Andrea Biondi

**Affiliations:** 1 Residency in Pediatrics, University of Milano Bicocca, Monza, Italy; 2 Laboratory for Mother and Child Health, Department of Public Health, Istituto Di Ricerche Farmacologiche Mario Negri IRCCS, Milan, Italy; 3 Department of Pediatrics, Fondazione IRCCS San Gerardo Dei Tintori, Monza, Italy; 4 Department of Medicine and Surgery, University of Milano Bicocca, Monza, Italy

**Keywords:** Bronchiolitis, Epidemic, Respiratory syncytial virus, Sars-CoV-2

## Abstract

**Background:**

The aim of this study is to compare the 2021–2022 bronchiolitis season to the four previous years (2017–2018, 2018–2019, 2019–2020, 2020–2021) to see if there was an anticipation of the peak, an overall increase of cases, and an increased need of intensive care.

**Methods:**

A retrospective single-centre study in the San Gerardo Hospital Fondazione MBBM, Monza, Italy was performed. Emergency Departments (ED) visits of patients aged < 18 years and ≤ 12 months were analyzed: the incidence of bronchiolitis on total assessments, the urgency level at triage and the hospitalization rate were compared. Data of children admitted to the Pediatric Department due to bronchiolitis were analyzed in terms of need of intensive care, respiratory support (type and duration), length of hospital stay, main etiological agent, patient characteristics.

**Results:**

During 2020–2021 (first pandemic period) an important reduction in the ED attendance for bronchiolitis was observed, while in 2021–2022 there was an increase in incidence of bronchiolitis (13% of visits in infants < 1 year) and in the rate of urgent accesses (p = 0.0002), but hospitalization rates did not differ compared to previous years. Furthermore, an anticipated peak in November 2021 was observed. In the 2021–2022 cohort of admitted children to the Pediatric Department, a statistically significative increased need of intensive care unit was detected (Odds Ratio 3.1, 95% CI 1.4–6.8 after adjustment for severity and clinical characteristics). Instead, respiratory support (type and duration) and length of hospital stay did not differ. RSV was the main etiological agent and RSV-bronchiolitis determined a more severe infection (type and duration of breathing support, intensive care need and length of hospital stay).

**Conclusions:**

During Sars-CoV-2 lockdowns (2020–2021), there was a dramatic decrease of bronchiolitis and others respiratory infections. In the following season, 2021–2022, an overall increase of cases with an anticipated peak was observed and data analysis confirmed that patients in 2021–2022 required more intensive care than children in the four previous seasons.

**Supplementary Information:**

The online version contains supplementary material available at 10.1186/s13052-023-01425-8.

## Background

Bronchiolitis is a major burden of the lower respiratory tract for infants and young children [1]. It is a seasonal infection, which typically presents between October and April in the northern hemisphere.

In March 2020, the World Health Organization (WHO) declared the Sars-CoV-2 outbreak a pandemic [2]. Because this virus is mainly airborne, measures such as social distancing, frequent hand washing and the usage of face-masks have been implemented worldwide. These actions influenced the transmission of other respiratory viruses [3]. A dramatic decrease of influenza and other respiratory infections, including bronchiolitis, has been globally described [4–7]. Italian Emergency Departments (ED) assisted a significant reduction of bronchiolitis admitted to the ED during the 2020–2021 cold season [8–10] and an overall reduction of acute respiratory tract infections [11] and hospitalized patients [12]. The second year of the pandemic has been characterized by a loosening of closure measures thanks to mass vaccination campaigns, allowing an increase in social interactions. To date, more than 90% of the Italian population over 12 years old has been vaccinated against Sars-CoV-2 [13]. Because of the increase of social interactions, a recurrence of common respiratory infections was expected. The 2021–2022 bronchiolitis surge was first described in Australian children [14], followed by intercontinental reports [15–18]. Camporesi et al. and Nenna et al. [19, 20] describe the anticipation of the bronchiolitis peak in Italy (October–November vs. mid-February), but they do not confirm an increase in overall severity. The aim of this study is to compare the 2021–2022 season to the four previous years (2017–2018, 2018–2019, 2019–2020, 2020–2021) to see if there was an anticipation of the peak, an overall increase of cases, and an increase of severity.

## Materials and methods

This is a single-centre retrospective study carried out at the San Gerardo Hospital—“Fondazione Monza e Brianza per il Bambino e la sua Mamma”, Monza, Italy. No ethics committee approval is required in Italy for epidemiological studies using health care administrative databases for research purposes and with individuals identified by an anonymous patient code.

Emergency Department (ED) admissions of children aged 0–18 years between September and April of 2017–2022 were analyzed, as well as children younger than 12 months old and with the clinical diagnosis of bronchiolitis (e.g., children with rhinorrhea, cough, crackles, wheezing, dyspnea, polypnea, feeding difficulties, apnea, lethargy) [21, 22]. There is no full agreement on the upper cut-off for diagnosing bronchiolitis, despite the majority account 12 months as the upper age limit [20–23], so this cut-off was considered in this study. The etiology of bronchiolitis was determined by nose swab or by rhino pharynx aspirate analyzed by Allplex™ Respiratory Panel Assays by Seegene Inc, an assay for the detection and identification of 26 pathogens (viruses and bacteria) using one-step real-time RT-PCR, or by ID NOW™ RSV PCR by Abbott.

In the ED a stratification of severity is obtained by assignment of a colour code by triage nurses, based on nationwide criteria: red (nondeferrable emergency, life-threatening condition), yellow (urgent, but not immediate life-threatening condition), green (low urgency and priority, deferrable care), and white code (nonurgent). Infants who received a diagnosis of bronchiolitis in the ED were either discharged, admitted to the Pediatric Department according to international and Italian guidelines [24], or transferred to another hospital due to full capacity. Need of intensive care was defined as the need of intensive respiratory support such as continuous positive airway pressure (CPAP) or mechanical ventilation. Risk factors were classified as prematurity (< 37 weeks gestational age at birth) and chronic disease (e.g. Down Syndrome, inborn errors of metabolism). No patients included in the study had congenital heart disease, because such patients are referred to other hospitals in Lombardy.

Only the first episode of bronchiolitis in children with more than one throughout the same season was considered.

During the 2017–2022 period, the overall capacity of the Pediatric Department has remained the same (14–17 hospital beds available per day).

The primary outcome of the study was to determine if the 2021–2022 bronchiolitis season was more severe in terms of need of intensive care in comparison to the seasons 2017–18, 2018–19, 2019–20, 2020–21.

The secondary outcomes were to compare the need of respiratory support and hospital stay length; to define the main etiological agents throughout the five different seasons; to compare disease severity in children with RSV versus non-RSV bronchiolitis; to compare disease severity in children with RSV and no risk factors (e.g., prematurity) versus RSV and risk factors.

Our hospital is situated in Monza, Lombardy, the first and most affected region in Italy during the Sars-CoV-2 outbreak. Lockdown was instituted between March and May 2020 and between October 2020 and March 2021 [25, 26]. Primary schools (6–11 years old) were closed from March to June 2020 and from mid-February to April 2021.

We ran the statistical analysis on R (4.1.2). For categorical variables, we compared groups by using the Fisher exact test for count data (when the variable took two values) or Pearson's Chi-squared test (when the variable could take more than 2 values). None of the continuous variables appeared to follow a normal distribution, which was tested by running the Shapiro Wilk Normality test. Because of this, discrete and continuous variables were tested using the Wilcoxon rank sum test. For all tests, the null was rejected at the 5% significance level. Missing data was omitted from the analysis. In the tables, each value's count is shown for categorical variables while the median and range is shown for discrete and continuous variables.

Univariate analyses were performed to evaluate the association between the admission to intensive care unit (primary outcome) and the following variables: period (2021–22 versus 2017–2020), age (≥ 60 vs. < 60 days), presence of risk factors (yes vs. no), respiratory syncytial virus (RSV) as etiology (yes vs. no), oxygen need (HNFC/CPAP yes vs. no) and Silverman score (≥ 2 vs. < 2). The 2020–21 period was not considered in this analysis because of the limited size sample due to the pandemic. A stepwise regression analysis was performed to investigate the association between admission to ICU and the above covariates.

## Results

**Fig. 1:**
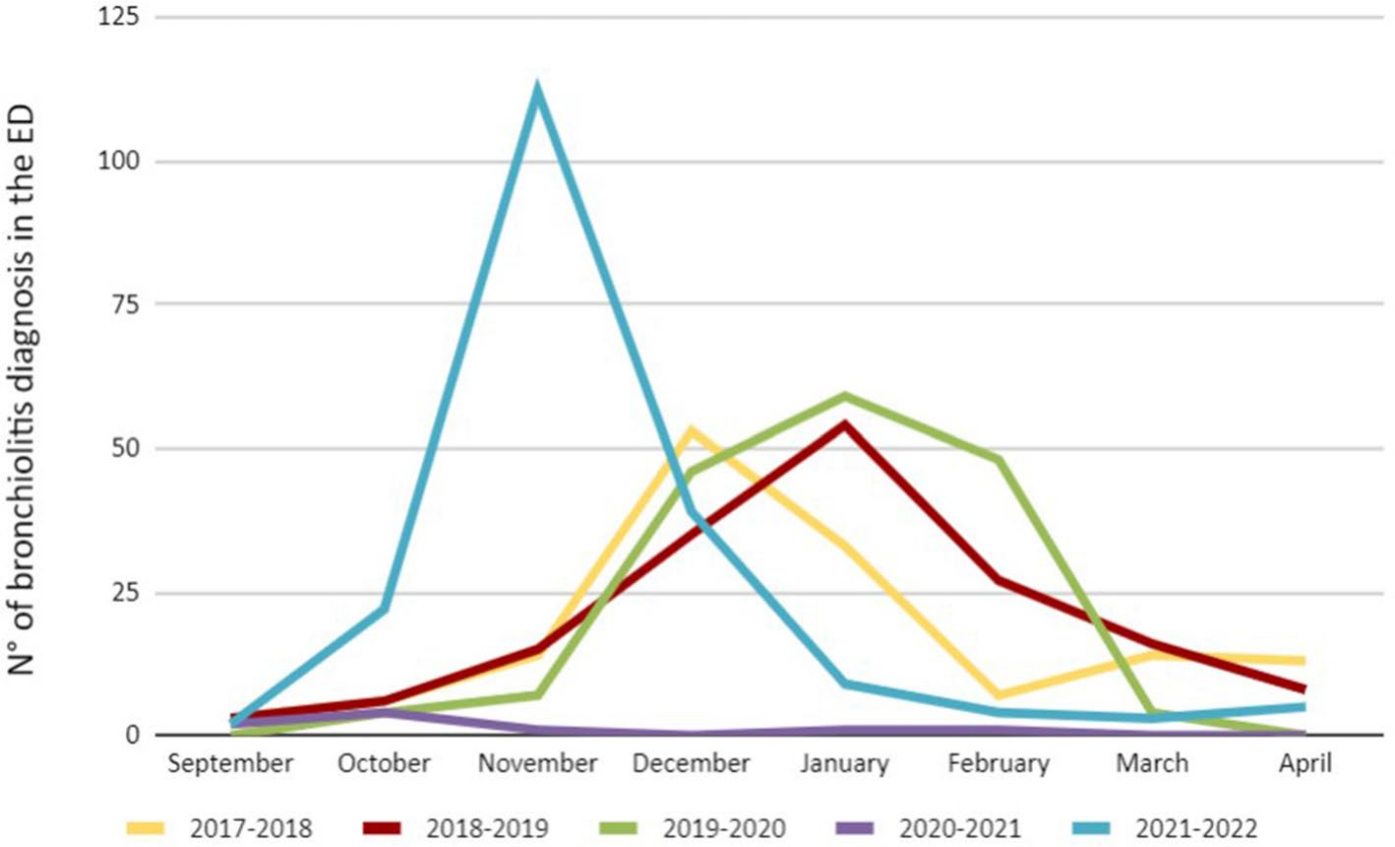
Emergency Department (ED) diagnosis of bronchiolitis in ≤ 1 year old children

Total admissions of children and adolescents to ED during the study period decreased from 15,194 in 2017–2018 to 12,335 in 2021–2022. As described worldwide, there was a significant reduction of hospital visits during the Sars-CoV-2 pandemic (5,379 accesses between September 2020 and April 2021). Among all admissions, bronchiolitis diagnosis consisted in about 1% of all diagnosis; among children aged 0–12 months, bronchiolitis represented 7–13% of pediatric ED accesses, except for the pandemic year (1.4%). An anticipated peak was observed in November 2022, counting for 57% of all cases of the 2021–2022 season (2 bronchiolitis in September 2021, 22 in October 2021, 112 in November 2021, 39 in December 2021, 9 in January 2022, 4 in February 2022, 3 in March 2022 and 5 in April 2022) (Fig. 1).

Overall, among children aged 0–12 months with a diagnosis of bronchiolitis, 15% was brought to the ED more than once, with no differences across seasons. In the 2021–2022 season there was a statistically significative difference in severity at presentation based on the colour code of assignment: 34% of bronchiolitis were given a red or yellow code in comparison to 19% of the previous years (χ2 = 18,5, *p* = 0.0002). Overall, 362 of 680 (53.2%) children with bronchiolitis at ED were hospitalized, 141 of whom were admitted to the Pediatric Department of San Gerardo hospital (Table 1). The hospitalization rate did not change throughout the five years (*p* = 0.25).

**Table 1:** Population characteristics of the cohorts of children hospitalized in the Pediatric Department due to bronchiolitis

	**2017–2018 *N* = 29**	**2018–2019 *N* = 25**	**2019–2020 *N* = 35**	**2020–2021 *N* = 2**	**2021–2022 *N* = 50**	**Total *N* = 141**
Age (days), median (min–max)	72 (25–338)	73 (37–358)	78 (22–247)	31 (27–34)	63 (22–316)	40 (22–358)
Risk factors (none/other disease/prematurity)	8/29 (21/2/6)	5/25 (20/2/3)	11/35 (24/4/7)	0/2 (2/0/0)	13/50 (37/6/7)	37/141 (104/14/23)
Silverman Score (0/1/2/3/unknown)	3/11/10/3/2	9/8/7/1/0	5/13/16/1/0	1/1/0/0/0	8/13/24/5/0	26/46/57/10/2
O2 Saturation, median (min–max)	93 (86–100)	92 (77–100)	95 (88–100)	98 (97–100)	95 (78–100)	89 (77–100)
Weight (kg), median (min–max)	5 (3.5–9)	5.5 (4–9.7)	5.25 (3.16–9.05)	4.75 (4–10.2)	4.9 (2.8–9.3)	4.2 (2.8–10.2)
Length of Admission (days), median (min–max)	7 (2–43)	7 (3–13)	6 (2–17)	3 (3–4)	7 (2–20)	6.5 (2–43)
Respiratory Support (none/O2 low flow/HFNC/CPAP)	27/29 (2/4/22/1)	19/25 (6/3/12/4)	28/35 (7/4/23/1)	1/2 (1/0/1/0)	42/50 (8/5/33/4)	116/141 (24/16/91/10)
Length of Respiratory Support, days, median (min–max)	5.5 (0–21)	6 (0–12)	4 (0–13)	0.5 (0–1)	6 (0–19)	5.5 (0–21)
Need of Intensive Care Unit, (%)	7/29 (24%)	4/25 (16%)	5/35 (14%)	0/2 (0%)	20/50 (40%)	36/141 (25%)
Need for nasogastric feeding	5/29	3/25	4/35	0/2	10/50	22/141
Need for intravenous fluids	12/29	17/25	13/35	0/2	15/50	57/141
Viral etiology (RSV/ non RSV/ unknown)	23/4/2	18/6/1	23/8/4	0/2/0	39/11/0	103/31/7

*HFNC* High Flow Nasal Cannulae, *CPAP* continuous positive airway pressure, *RSV* Respiratory Syncytial Virus

There was an increased need of intensive care in 2021–2022 in comparison to the 2017–21 period (*p* = 0.005). Instead, no statistically significative differences were found in terms of severity at presentation (Silverman Score and peripheral oxygen saturation, *p* = 0.35 and *p* = 1 respectively), respiratory support type and duration (*p* = 0.96 and *p* = 0.55), length of stay (*p* = 0.73), nasogastric feeding or intravenous hydration (*p* = 0.33 and *p* = 0.07), neither were there no differences in terms of age at presentation (63 vs 72 days, *p* = 0.13), weight (4.9 vs. 5.2 kg, *p* = 0.23), etiology (*p* = 1, RSV was the main viral agent) and presence of risk factors (*p* = 0.74).

No statistically significant difference was observed also when comparing the characteristics of 2021–2022 cohort versus 2018–19 (before the beginning of the pandemic), except for intensive care admission (*p* = 0.04) and need of intravenous hydration (*p* = 0.003). In 2021–2022 no deaths occurred, and no short-term sequelae are reported.

At the univariate analysis, the likelihood of being admitted to ICU was influenced by observation period, presence of risk factors, need of HFNC/CPAP, RSV as causal agent and Silverman Score. After adjustment of the above covariates, a greater likelihood of ICU admission was observed in 2021–22 period versus 2017–2020 (aOR 3.6; 95%,CI 1.5–8.9) (Table 2). In the analysis of the five cohorts (2017–2022), RSV was the main etiological agent (Table 2) (79% in 2017–2018, 72% in 2018–2019, 66% in 2019–2020, 0% in 2020–2021, 78% in 2021–2022). The children with RSV-bronchiolitis, throughout the entire study period (2017–2022) required more oxygen support (length and type), intensive care, and longer hospital stay in comparison to those with RSV negative bronchiolitis (Supplementary Table 1).

**Table 2:** Analysis of the association between admission to intensive care unit, patient characteristics, bronchiolitis etiology, need of respiratory support and observation period

**Variable**		**Admitted to Intensive care unit (*N* = 36)**	**Total number of children hospitalized for bronchiolitis 2017–2022 (*N* = 141)**	**Odds Ratio**	***p*-value**	aOR*	***p*-value**
Period	*2021–22*	20 (40.0)**	50	3.1 (1.4–6.8)	0.005	3.6 (1.5–8.9)	0.005
	*2017–20*	16 (17.6)	91	1		1	
Age (days)	> *60*	18 (21.4)	84	0.6 (0.3–1.3)	0.24	n.s	n.s
	≤ *60*	18 (31.6)	57	1			
Risk factors	*Yes*	17 (44.7)	38	3.6 (1.6–8.0)	0.002	4.4 (1.7–11.3)	0.002
	*No*	19 (18.5)	103	1			
RSV***	*Yes*	33 (32.0)	103	4.4 (1.2–15.5)	0.02	5.4 (1.3–22.2)	0.02
	*No*	3 (9.7)	31	1		1	
HFNC/CPAP	*Yes*	34 (33.7)	101	9.6 (2.2–42.4)	0.0002	8.0 (1.6–39.1)	
	*No*	2 (5.0)	40	1			
Silverman Score	≥ *2*	25 (37.3)	67	3.7 (1.6–8.5)	0.002	n.s	n.s
	< *2*	10 (13.9)	72	1			

^*^ aOR: Odds Ratio adjusted for other covariates

^**^rate of admission to ICU (N° of children admitted/total number of hospitalized children*100) is given in bracket

^***^ for 7 cases the etiological agent was unknown (none admitted to ICU)

Finally, we compared the severity of the disease of children with RSV-bronchiolitis and risk factors (e.g. prematurity, other diseases) versus children with RSV-bronchiolitis and no risk factors between 2017 and 2022, confirming an increased need of intensive care (*p* = 0.001) and nose-tube feeding (*p* = 0.001) for children with RSV bronchiolitis and risk factors, and older age at time of presentation (*p* = 0.001). There were no differences between these two cohorts in terms of oxygen blood peripheral saturation (*p* = 0.88), Silverman score (*p* = 0.73), type of respiratory support (*p* = 0.88), length of respiratory support in days (*p* = 0.2), length of hospital stay (*p* = 0.22) and need of intravenous fluids (*p* = 0.81).

## Discussion

During the Sars-CoV-2 pandemic, a dramatic decrease in viral infections has been described in the pediatric population [11], due to social-distancing, closure of schools, implementation of hand washing and the usage of face-masks [4, 5, 7, 27]. This phenomenon included bronchiolitis and has been described in Italy as well [8, 9], and the present findings confirm what observed between September 2020 and April 2021.

During the summer of 2021, alerts were released concerning an outbreak of an abnormal bronchiolitis season in terms of overall numbers and distribution. It was initially anticipated by pediatricians in the Southern Hemisphere [18], followed by worldwide studies, which reported an increase in overall incidence and an anticipation of the usual peak [6, 15–17]. In our results, after a disappearance of bronchiolitis during the first lockdown, an increase in overall incidence during September 2021 and April 2022 was observed. Moreover, this study confirms the anticipated peak and shorter duration of the season described by other Italian reports [19, 20] (Fig. 1).

In the 2021–2022 season, there was a double increase in severity at presentation based on the colour of assignment from triage nurses at admission in ED. Even so, the admission rate did not change throughout the five years.

Guitart et al. [16] describe a more severe 2021 bronchiolitis season in comparison to the previous ten years in terms of length of stay and need of pediatric intensive care unit. Whereas, in Italy, Camporesi et al. [19] do not confirm an unusual disease severity. In our sample, a three time increase after adjustment was observed in intensive care admission during 2021–2022 season in agreement with Guitart et al. findings [16]. It is not possible to completely exclude that the increase in ICU admission in 2021/22 may be due to different criteria adopted by health professionals, but in our opinion it is unlikely, since the availability of beds was scant.

In our study RSV was the main etiological agent as reported in literature [1, 21, 22], except for 2020–2021. Bronchiolitis due to RSV resulted more severe in terms of respiratory support, intensive care and length of hospital stay in comparison to those with RSV negative bronchiolitis. In literature, there is no consensus whether this is true or not [20, 22, 28, 29]. An increase of intensive care accesses and nose-tube feeding, and older age at time of presentation was observed in children with RSV-bronchiolitis and risk factors (e.g. prematurity, other diseases), in accordance with previously reported data [24, 30, 31].

The abnormal bronchiolitis peak may be explained by a global loosening of Sars-CoV-2 prevention measures and an increase in social interactions, in association to a waned herd immunity to other viruses and an increase in RSV-naïve patients. In fact, children affected by bronchiolitis during 2021–2022 were born during the Sars-CoV-2 pandemic, with an immune system that was less stimulated by external agents (bacteria, virus and others) due to all the Sars-CoV2 restrictions; even their mothers were not exposed to infections and did not develop antibodies. Furthermore, Nenna et al. described an opposite hospital admission trend between RSV and Sars-CoV-2 during their study period (2021–2022) confirming a close relationship between these two viruses [20]. A hypothesis which has been addressed is that of “viral interference”: if a person is co-infected by two viruses at the same time, the immune responses toward one of them reduces the possibility to replicate of the other [16, 32, 33]. Another hypothesis that should be considered is that during the Sars-CoV-2 pandemic there may have been a genetic lineage shift in RSV, as previously observed [34]. Finally, an association with weather and air pollution has been hypothesized: climate factors could possibly influence RSV spread, as Nenna et al. [35] suggest on a ten-year study on Italian infants with RSV bronchiolitis before Sars-CoV2 outbreak. Manti et. al [36] argue this hypothesis as well during the 2020 lockdown, during which a 60% decrease of air pollutants was observed, as well as a reduction of RSV bronchiolitis. Further studies are needed.

Analysis of future seasons will allow a better understanding of the impact of Sars-CoV-2 on respiratory infections. Recent reports from the Southern Hemisphere and the United Kingdom describe abnormal Influenza and RSV seasons in terms of incidence and distribution of cases [37–40].

The limitations of this study are that it is a retrospective single centre study with a limited sample size. The diagnosis of bronchiolitis is clinical; few patients did not have a defined etiological agent. In those without an identified germ, extensive viral testing was not performed. However, the etiological agent, according to Italian and international guidelines, does not affect treatment.

## Conclusions

During Sars-CoV-2 lockdowns (2020–2021), there was a dramatic decrease of ED assessments and a dramatic decrease of clinical diagnosis of bronchiolitis, followed by an anticipated and significantly increased peak of bronchiolitis during the 2021–2022 season. Children with bronchiolitis assessed in the ED were triaged with more urgent codes, but hospitalization rates did not change. However, admissions to the Intensive Care Unit were higher than in the four previous investigated seasons. Clinicians should prepare for abnormal RSV seasons, in terms of incidence, distribution and severity.

## Supplementary Information

**Additional file 1: Supplementary Table 1.** Comparison between the RSV-bronchiolitis and non-RSV bronchiolitis in the 2017-2022 study period.

## Data Availability

The datasets used and/or analyzed during the current study are available from the corresponding author on reasonable request.

## Abbreviations

ED: Emergency Departments
Sars-CoV-2: Severe Acute Respiratory Syndrome Coronavirus—2
BPD: Bronchopulmonary Dysplasia
RSV: Respiratory Syncytial Virus
HFNC: High Flow Oxygen on Nasal Cannulae
CPAP: Continuous Positive Airway Pressure
RT-PCR: Reverse Transcriptase-Polymerase Chain Reaction
