# Supplementary material for: The bronchiolitis epidemic in 2021–2022 during the SARS-CoV-2 pandemic: experience of a third level centre in Northern Italy
Source: Ital J Pediatr. 2023 Feb 21;49:26. doi: 10.1186/s13052-023-01425-8 (PMC9942300; doi:10.1186/s13052-023-01425-8)
Supplement: Supplementary file 1 — Additional file 1: Supplementary Table 1. Comparison between the RSV-bronchiolitis and non-RSV bronchiolitis in the 2017-2022 study period. [file 13052_2023_1425_MOESM1_ESM.docx]

**Supplementary Table 1**. Comparison between RSV-bronchiolitis and non-RSV bronchiolitis in the 2017-2022 study period.

|  | **RSV-negative bronchiolitis**  **2017-2022**  **N=31** | **RSV-bronchiolitis**  **2017-2022**  **N=103** | **Total**  **N=134**  **N=7 unknown etiology** | **p-value** |
| --- | --- | --- | --- | --- |
| Age (days), median (min-max) | 89 (27-316) | 64 (22-358) | 40.5 (22-358) | 0.226 |
| Weight (kg), median (min-max) | 5.2 (3.34-9.7) | 5 (2.8-9.7) | 4.2 (2.8-9.7) | 0.316 |
| Risk Factors (None/Other disease/Prematurity) | 21/4/6 | 78/10/15 | 99/14/21 | 0.567 |
| O2 Saturation, median (min-max) | 95 (85-100) | 93 (77-100) | 89.5 (77-100) | 0.10 |
| Silverman Score (0/1/2/3/Unknown) | 6/14/9/2 | 15/31/47/8/2 |  | 0.316 |
| Respiratory Support  (none/O2 low flow/HFNC/CPAP or Helium) | 10/3/17/1 | 9/12/73/9 | 19/15/90/10 | 0.01 |
| Length of Respiratory Support, days, median (min-max) | 3(0-16) | 6 (0-21) | 5.5 (0-21) | 0.001 |
| Length of Admission (days), median (min-max) | 5 (2-16) | 7 (2-43) | 6.5 (2-43) | 0.008 |
| Need of Intensive Care Unit, (Yes/No, %) | 3/28/ (10%) | 33/70 (32%) | 36/98 (27%) | 0.019 |
| Need for nasogastric feeding, (Yes/No, %) | 2/29/ (6%) | 20/83/ (19%) | 22/112 (16%) | 0.103 |
| Need for intravenous fluids, (Yes/No, %) | 14/17 (45%) | 41/62 (39%) | 55/79 (41%) | 0.67 |

*HFNC= High Flow Nasal Cannulae; CPAP= continuous positive airway pressure; RSV=Respiratory Syncytial Virus*
